# Engineering Rare Earth-Assisted Cobalt Oxide Gels Toward Superior Energy Storage in Asymmetric Supercapacitors

**DOI:** 10.3390/gels11110867

**Published:** 2025-10-29

**Authors:** Pritam J. Morankar, Rutuja U. Amate, Aviraj M. Teli, Aditya A. Patil, Sonali A. Beknalkar, Chan-Wook Jeon

**Affiliations:** 1 School of Chemical Engineering, Yeungnam University, 280 Daehak-ro, Gyeongsan 38541, Republic of Koreaaditya.nanotechnology@gmail.com (A.A.P.); 2 Division of Electronics and Electrical Engineering, Dongguk University-Seoul, Seoul 04620, Republic of Korea

**Keywords:** cobalt oxide gels, rare earth doping, pseudocapacitance, asymmetric supercapacitor, sol–gel synthesis, high energy density, cycling stability, charge-transfer kinetics

## Abstract

The rational design of transition metal oxides with tailored electronic structures and defect chemistries is critical for advancing high-performance supercapacitors. Herein, we report the engineering of cobalt oxide (Co_3_O_4_) gels through controlled sol–gel synthesis and rare earth (RE) incorporation using neodymium (Nd), gadolinium (Gd), and dual neodymium/gadolinium (Nd/Gd) doping. X-ray diffraction (XRD) confirmed the preservation of the cubic spinel structure with systematic peak shifts and broadening, evidencing lattice strain, oxygen vacancy generation, and defect enrichment. Field-emission scanning electron microscopy (FE-SEM) analyses revealed distinct morphological evolution from compact nanoparticle assemblies in pristine Co_3_O_4_ to highly porous, interconnected frameworks in Nd/Gd–Co_3_O_4_ (Nd/Gd-Co). X-ray photoelectron spectroscopy (XPS) verified the stable incorporation of RE ions, accompanied by electronic interaction with the Co–O matrix and enhanced oxygen defect states. Electrochemical measurements demonstrated that the Nd/Gd–Co electrode achieved a remarkable areal capacitance of 25 F/cm^2^ at 8 mA/cm^2^, superior ionic diffusion coefficients, and the lowest equivalent series resistance (0.26 Ω) among all samples. Long-term cycling confirmed 84.35% capacitance retention with 94.46% coulombic efficiency after 12,000 cycles. Furthermore, the asymmetric pouch-type supercapacitor (APSD) constructed with Nd/Gd–Co as the positive electrode and activated carbon as the negative electrode delivered a wide operational window of 1.5 V, an areal capacitance of 140 mF/cm^2^, an energy density of 0.044 mWh/cm^2^, and 89.44% retention after 7000 cycles. These findings establish Nd/Gd-Co gels as robust and scalable electrode materials and demonstrate that RE co-doping is an effective strategy for bridging high energy density with long-term electrochemical stability in asymmetric supercapacitors.

## 1. Introduction

Modern energy storage is increasingly dictated by the interplay between electrode chemistry and device architecture, rather than by device type alone [1,2]. The decisive factor lies in designing materials that can simultaneously accommodate high charge density, rapid ion transport, and long-term structural stability under continuous cycling [3,4,5]. In this context, pseudocapacitive transition metal oxides (TMOs) have emerged as an exciting class of electrode materials, where charge storage occurs not only through electrostatic accumulation at the electrode–electrolyte interface but also via fast and reversible redox reactions [6,7]. Such mechanisms provide a pathway to achieve higher energy densities than purely double-layer capacitors while maintaining the high-power delivery and cycling durability required for next-generation energy storage [8,9]. Realizing this potential, however, demands precisely engineered TMOs that can overcome their inherent drawbacks of low electronic conductivity and sluggish reaction kinetics [10].

Among the wide variety of TMOs, materials such as MnO_2_, NiO, WO_3_, and V_2_O_5_ have been widely studied for supercapacitor electrodes due to their multiple oxidation states and high theoretical capacitances [11,12,13]. Despite these merits, their poor conductivity and structural instability under extended cycling hinder their practical application. Co_3_O_4_ distinguishes itself in this group owing to its natural abundance, environmental benignity, well-defined Co^2+^/Co^3+^ redox couples, and exceptionally high theoretical capacitance (~3560 F/g) [14]. Several studies have demonstrated its potential: Priyadharsini et al. [15] reported Co_3_O_4_ nanoparticles synthesized by a urea-assisted sol–gel method, forming a cubic structure with ~13–15 nm size. In 3 M KOH, the electrode delivered a high capacitance of 761.25 F/g. Manteghi et al. [16] demonstrated that nano Co_3_O_4_ (<40 nm), obtained from a cobalt–oxalate precursor with CTAB/F-127 surfactants and calcination, exhibited 351 F/g at 0.85 A/g in 2 M KOH with 98.6% retention after 1000 cycles on Ni foam. Jang et al. [17] synthesized porous Co_3_O_4_ nanorods hydrothermally, where morphology was tuned by calcination temperature. Electrochemical tests in 6 M KOH showed 226.3 F/g at 10 mV/s and ~76% retention after 5000 cycles for rods calcined at 300 °C. Yet, pristine Co_3_O_4_ still faces persistent challenges: sluggish ion diffusion, poor electrical conductivity, and structural degradation during cycling. These limitations motivate the exploration of electronic structure engineering and defect modulation strategies to enhance its electrochemical performance [6].

Elemental doping has proven to be an efficient approach for tailoring the physicochemical properties of TMOs. In particular, RE doping has emerged as a powerful strategy, owing to the unique electronic configuration of RE cations [18]. Their partially filled 4f orbitals strongly interact with the d orbitals of transition metals, effectively tuning the band structure and enhancing redox activity. Moreover, the relatively large ionic radii of RE ions introduce lattice strain and oxygen vacancies, thereby improving ionic transport and exposing additional electroactive sites [19]. Specifically, neodymium (Nd) doping facilitates enhanced electronic hybridization and conductivity, while gadolinium (Gd) incorporation promotes lattice distortion and defect generation, accelerating charge transfer [20,21,22]. A co-doping strategy that integrates Nd and Gd is expected to combine these complementary effects, yielding superior conductivity, structural stability, and pseudocapacitive behavior. Several prior reports substantiate this concept: Yang et al. [23] highlighted the effect of Pr_2_O_3_ doping in Co_3_O_4_ nanoflakes grown on Ni foam by hydrothermal synthesis. The electrode achieved 640 C/g at 2 A/g with 98% retention after 5000 cycles, and an ASC delivered 42.3 Wh/kg at 240 W/kg. Theerthagiri et al. [24] presented RE (La, Nd, Gd, Sm)-doped Co_3_O_4_ nanocapsules prepared by a polymer-assisted combustion method. Sm–Co_3_O_4_ showed the best performance with high capacitance and 93.18% retention after 5000 cycles. Israr et al. [25] revealed that Co–CeO_2_/C composites obtained from MOF precursors by pyrolysis exhibited excellent performance. The 5Co–5Ce/C electrode delivered 839 F/g at 0.1 A/g with 97% retention after 6000 cycles, due to synergistic Co–Ce interaction and mesoporosity. Furthermore, recent advances in cobalt oxides and cobalt mixed metal oxides have demonstrated their significant potential for supercapacitor applications. Cobalt ferrite and other mixed metal oxides anchored on conductive supports such as graphene oxide show enhanced electrochemical performance by improving conductivity and structural stability [26]. Facile synthesis methods for ternary graphene oxide-supported metal-doped cobalt oxide nanostructures have been reported to deliver high capacitance and excellent cycling durability due to synergistic effects between cobalt and dopant metals [27]. Moreover, graphene-metal oxide nanocomposites have emerged as promising electrodes by combining high surface area and efficient charge transfer, critical for next-generation energy storage devices [28]. These studies collectively underscore the importance of cobalt-based mixed oxides and their composites as versatile platforms for enhancing supercapacitor performance.

In this context, integrating RE dopants within a Co_3_O_4_ framework offers an effective avenue for engineering lattice distortion, enhancing electron transport, and facilitating reversible redox activity by modulating orbital hybridization and defect chemistry. Such RE-induced alterations not only stabilize the crystalline network during prolonged cycling but also expand the availability of electroactive sites, significantly improving charge diffusion and storage capacity. Motivated by these factors, the present study systematically explores the effect of Nd, Gd, and dual Nd/Gd doping on the structural, electronic, and electrochemical characteristics of Co_3_O_4_ gels synthesized via a sol–gel route. The sol–gel process enabled uniform dopant incorporation at the molecular level, yielding porous, interconnected networks favorable for ion accessibility and charge storage. The work aims to elucidate how the synergistic influence of co-doping influences crystallinity, morphology, and defect formation, thereby promoting superior capacitive performance and cycling stability. This co-doping approach introduces a new pathway for optimizing spinel-type cobalt oxides for high-performance. The prepared samples were extensively characterized by XRD to confirm crystallinity, FE-SEM to evaluate morphology and nanostructure, and XPS to analyze chemical states, oxygen vacancies, and dopant incorporation. Electrochemical investigations, including cyclic voltammetry (CV), galvanostatic charge–discharge (GCD), and electrochemical impedance spectroscopy (EIS), were carried out in a three-electrode configuration, followed by assembly of an APSD using activated carbon as the negative electrode. Ultimately, this research provides a rational strategy for developing RE-engineered Co-based oxides as high-performance electrode materials for asymmetric supercapacitor applications, bridging the gap between high power capability and practical energy density.

## 2. Results and Discussion

### 2.1. X-Ray Diffraction Elucidation

**Figure 1:**
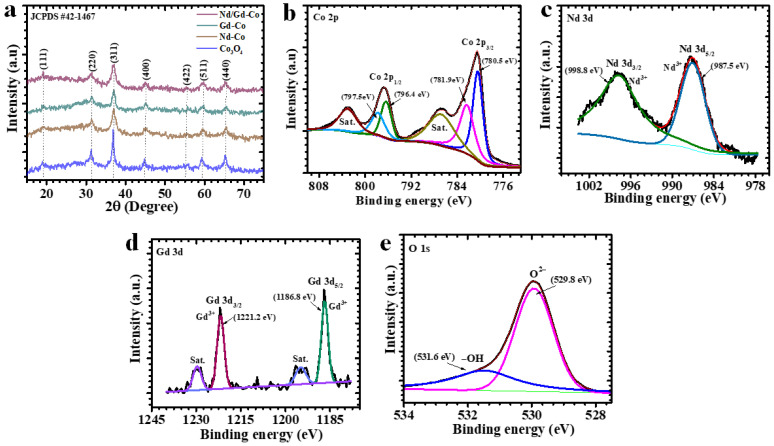
(**a**) XRD pattern of all electrodes confirming the spinel phase, XPS spectra of the Nd/Gd-Co electrode showing (**b**) Co 2p (**c**) Nd 3d (**d**) Gd 3d and (**e**) O 1s core levels.

The crystalline structures of pristine Co_3_O_4_ and RE-Co_3_O_4_ were examined by XRD, as shown in Figure 1a. All of the diffraction peaks can be assigned to the cubic spinel phase of Co_3_O_4_ (JCPDS No. 42-1467), with reflections at 2θ ≈ 19.0°, 31.3°, 36.8°, 44.8°, 59.3°, and 65.2°, corresponding to the (111), (220), (311), (400), (511), and (440) planes. The absence of extra peaks related to impurity phases confirms that both pristine and doped samples are phase-pure. When compared with pristine Co_3_O_4_, the RE–Co_3_O_4_ electrodes retain the same spinel framework, suggesting that Nd and Gd ions enter the Co_3_O_4_ lattice rather than forming separate phases. A small shift in the peak positions is evident in the doped samples, which can be attributed to lattice strain caused by substitution of Co with the larger RE cations. This lattice distortion alters the unit cell parameters and is often accompanied by the generation of oxygen vacancies that are known to improve redox activity [15]. A noticeable decrease in peak intensity and broadening of the reflections is also observed in the doped electrodes. These features point to increased structural disorder, which is usually advantageous for electrochemical applications since it provides more accessible surface sites and facilitates ion transport. Importantly, the more pronounced distortion in the Nd/Gd co-doped sample suggests a synergistic effect, where the combined ionic radii and electronic configurations of both rare earth dopants introduce a higher concentration of structural defects and strain compared to single doping. These defects serve as rapid ion diffusion channels and additional redox-active centers, thus justifying the improved electrochemical performance observed experimentally [29]. Moreover, quantitative XRD analysis was performed to evaluate the lattice parameters and average crystallite sizes of all synthesized samples. The lattice parameter *a* for each sample was calculated for cubic spinel Co_3_O_4_-type structures using Equation (1) [30]:

(1) $$a=d_{hkl}\sqrt{h^{2}+k^{2}+l^{2}}$$

where the interplanar spacing *d_hkl_* was obtained from Bragg’s law (2):

(2) $$d_{hkl}=\frac{\lambda }{2sin\theta }$$

with *λ* = 1.5406 Å (Cu Kα), and *θ* is half of the measured 2*θ* peak position.

The crystallite size *D* was estimated using the Scherrer Equation (3) [31]:

(3) $$D=\frac{K\lambda }{\beta cos\theta }$$

where *K* is the shape factor (0.9), *λ* is the X-ray wavelength, *β* is the FWHM in radians, and *θ* is the Bragg angle. The calculated average lattice parameters and crystallite sizes are summarized Table S1. These results indicate that rare earth doping induces subtle reductions in lattice parameter and crystallite size, consistent with local lattice distortion and microstructural refinement introduced by Nd and Gd incorporation. Taken together, the XRD results confirm the effective incorporation of Nd and Gd into the spinel structure and highlight that co-doping produces greater lattice distortion and defect generation, thereby creating a more favorable structural environment for enhanced pseudocapacitive performance [16,17].

### 2.2. X-Ray Photoelectron Spectroscopy Elucidation

The high-resolution XPS spectra of the Nd/Gd–Co electrode are presented in Figure 1b–e. These spectra provide clear insight into the oxidation states of cobalt, the oxygen environment, and the successful incorporation of the RE dopants. In the Co 2p spectrum (Figure 1b), two main spin–orbit peaks are observed at Co 2p_3/2_ and Co 2p_1/2_, together with their associated satellite features. The fitted spectrum shows the coexistence of Co^2+^ and Co^3+^, which is consistent with the spinel nature of the oxide. A small shift in the binding energies is evident, suggesting that the local electronic environment of cobalt is slightly modified by the presence of Nd and Gd in the lattice. This indicates that co-doping alters the coordination state of cobalt without disturbing its redox-active nature [32]. The Nd 3d spectrum (Figure 1c) shows two distinct spin–orbit doublets, confirming the trivalent state of neodymium. A slight change in the binding energy compared with typical Nd^3+^ reference points to an electronic interaction between Nd ions and the surrounding cobalt–oxygen framework. Similarly, the Gd 4d spectrum (Figure 1d) displays well-resolved features characteristic of Gd^3+^, again with a minor shift in peak position that can be related to lattice distortion caused by gadolinium incorporation. The absence of additional peaks in both cases indicates that the RE dopants are stably incorporated without forming secondary phases [2]. The O 1s spectrum (Figure 1e) can be divided into three main contributions. The low-binding-energy component corresponds to lattice oxygen bound to cobalt in the crystalline network. The second contribution, appearing at slightly higher binding energy, arises from oxygen associated with vacancy sites and structural defects. These sites are important because they act as channels for ionic migration and enhance electronic conductivity. The third contribution, at higher binding energy, is linked to surface-adsorbed species such as hydroxyl groups or chemisorbed oxygen. A subtle shift in the O 1s peak positions, along with the clear presence of the vacancy-related component, indicates that Nd and Gd incorporation generates lattice strain and defect states, thereby increasing the density of active oxygen sites. Overall, the XPS results confirm that the Nd/Gd–Co electrode retains a mixed Co^2+^/Co^3+^ valence system, while co-doping introduces oxygen vacancies and modifies the electronic structure of the lattice. The slight shifts in binding energies across Co, O, Nd, and Gd spectra provide further evidence of lattice distortion and strong dopant–host interaction. Such features are directly linked to improved charge-transfer properties and enhanced electrochemical activity, which explain the superior performance of the Nd/Gd–Co electrode [33,34]. Overall, the characteristic binding energies and area percentages for each major fitted peak are summarized in Table S2. Relative area percentages were calculated for each component to quantitatively assess the surface chemical states, confirming the coexistence of Co, Nd, and Gd species and substantial oxygen incorporation. This combined analysis verifies successful doping and the multicomponent nature of the prepared samples.

### 2.3. Morphological and Elemental Compositional Characteristics

**Figure 2:**
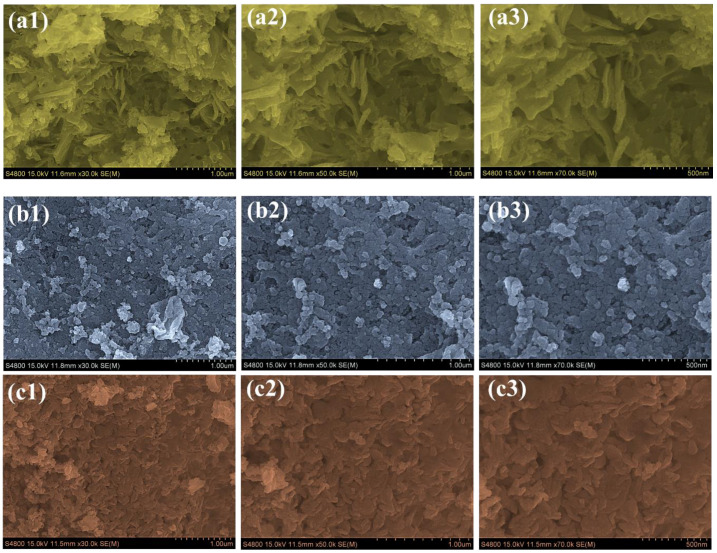
FE-SEM images at various magnifications of (**a1**–**a3**) Nd–Co (**b1**–**b3**) Gd–Co and (**c1**–**c3**) Nd/Gd–Co samples, illustrating morphological evolution.

The FESEM images reveal distinct morphological differences between pristine Co_3_O_4_ and RE-Co_3_O_4_ electrodes, highlighting the impact of RE incorporation on the surface texture and particle assembly. The FESEM images of pristine Co_3_O_4_ (Figure S1a–c) show a compact surface formed by agglomerated nanoparticles. These particles are closely packed and appear as dense clusters with limited porosity. Such a morphology is relatively rigid but leaves little open space between particles. When Nd is introduced, the Nd-Co sample (Figure 2(a1–a3)) develops a rougher and more porous arrangement. The surface displays loosely stacked, flake-like features along with an uneven texture. Compared with pristine Co_3_O_4_, the Nd-Co morphology exhibits greater surface roughness and wider interparticle spacing, which indicates a clear tendency toward enhanced porosity. In the case of Gd doping, the Gd-Co sample (Figure 2(b1–b3)) shows a distinct morphological change compared with pristine Co_3_O_4_. The particles are not as tightly aggregated, and the overall surface appears more open. This suggests that Gd incorporation disrupts the dense packing of the pristine oxide, producing a less compact structure with higher surface exposure. The most striking change is observed in the Nd/Gd–Co electrode (Figure 2(c1–c3)). Here, the surface evolves into a highly porous and interconnected network. The particles organize into a mixed architecture, combining fine grains and flake-like domains that are uniformly distributed. This results in a well-developed hierarchical structure with open channels and a three-dimensional framework, which is significantly different from the compact clusters of pristine Co_3_O_4_. These progressive morphological changes strongly influence the expected electrochemical behavior. The compact and agglomerated surface of pristine Co_3_O_4_ restricts ion diffusion and limits the number of accessible electroactive sites. Nd doping improves the surface by introducing greater porosity and roughness, while Gd doping alleviates aggregation and produces a more open structure. However, the Nd/Gd–Co electrode provides the most beneficial morphology overall. Its interconnected and porous framework offers abundant electroactive sites, facilitates rapid ion transport, and provides better structural stability during repeated charge–discharge cycles. Therefore, the Nd/Gd–Co electrode is expected to deliver the best performance among all the samples, making it particularly suitable for high-energy and stable supercapacitor applications.

**Figure 3:**
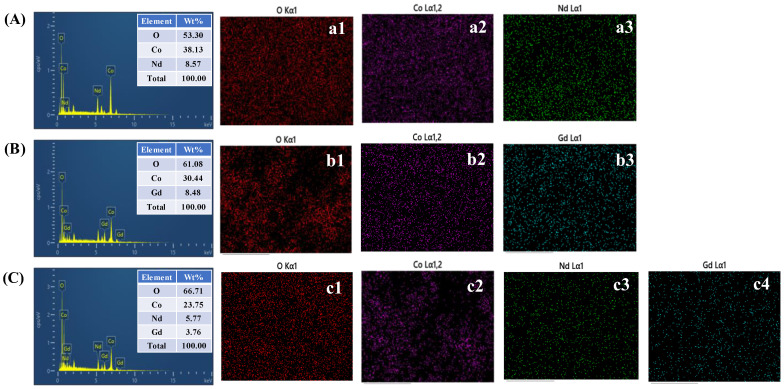
EDS spectra and corresponding mapping analysis images of (**A**–**a3**) Nd–Co (**B**–**b3**) Gd–Co and (**C**–**c4**) Nd/Gd–Co samples.

The EDS spectra together with the corresponding elemental mapping images provide clear insight into the composition and distribution of elements in all the samples, as shown in (Figure 3(A–c4)) and Figure S1d–f. For pristine Co_3_O_4_, shown in Figure S1d–f, only cobalt and oxygen signals are detected, and the mapping confirms their uniform distribution across the structure, demonstrating the high purity of the material. When Nd or Gd is introduced, additional peaks corresponding to the dopant elements appear in the spectra, and the elemental maps confirm their homogeneous dispersion together with cobalt and oxygen. In the case of the Nd/Gd-Co electrode, both Nd and Gd are simultaneously detected, and the mapping images verify that these dopants are uniformly distributed alongside cobalt and oxygen throughout the electrode. The absence of clustering or localized enrichment indicates that the sol–gel route enabled efficient incorporation of the RE ions into the oxide matrix. Such uniform elemental distribution is essential because it ensures consistent structural modification and homogeneous electrochemical activity. Among all the samples, the Nd/Gd-Co electrode exhibits the most balanced and well-dispersed elemental distribution, which provides enhanced ion accessibility, promotes defect generation, and strengthens redox activity. This explains why the co-doped system shows the most favorable morphology composition relationship for achieving superior electrochemical performance.

### 2.4. Electrochemical Results and Analysis

**Figure 4:**
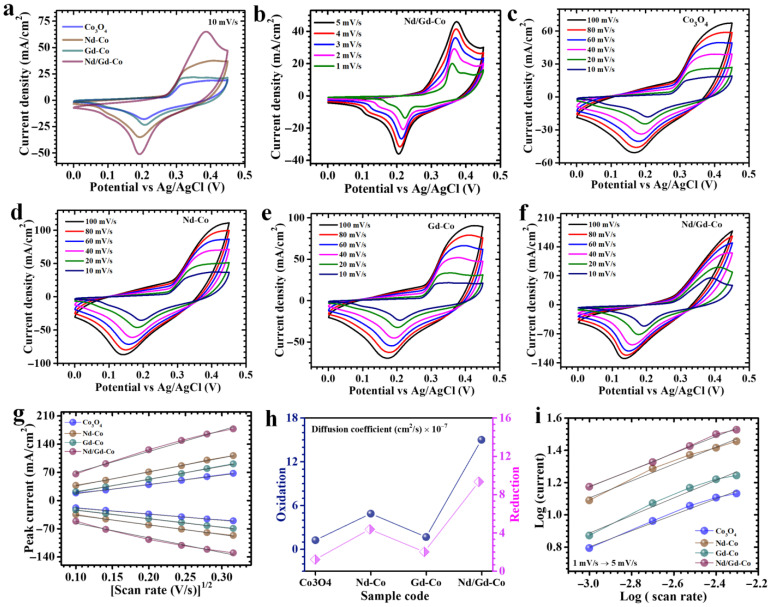
Cyclic voltammetry of (**a**) pristine Co_3_O_4_ and RE-Co_3_O_4_ electrodes at a scan rate of 10 mV/s, (**b**) CV of Nd/Gd–Co electrode at lower scan rate of 1–5 mV/s, (**c**–**f**) CV of pristine and RE-Co_3_O_4_ electrodes at a scan rate of 100 mV/s, (**g**) plot of peak current vs. (scan rate)^1/2^, (**h**) graphical presentation of calculated diffusion coefficients, and (**i**) Plot of *log*(*i*) against the *log*(*ϑ*), indicating charge storage behavior mechanisms.

The CV characteristics of pristine and RE-modified Co_3_O_4_ electrodes clearly demonstrate the strong coupling between structural tailoring and electrochemical activity. As shown in Figure 4a, all electrodes deliver well-resolved anodic and cathodic features within 0.0–0.45 V (vs. Ag/AgCl) at a 10 mV/s scan rate, centered around ~0.32 V and ~0.18 V, respectively. These signatures reflect the reversible Co^2+^/Co^3+^ transitions that are sustained through the continuous exchange of hydroxyl ions with the alkaline electrolyte, a character of surface-controlled pseudocapacitance. Notably, the Nd/Gd-Co electrode achieves the broadest CV area and the sharpest redox responses, surpassing both the pristine and single-doped counterparts. This enhancement is not a trivial increase in capacity but a demonstration of synergistic RE substitution. The simultaneous incorporation of Nd^3+^ and Gd^3+^ ions introduce controlled lattice strain and defect states, which (i) modulate the electronic configuration of cobalt centers, (ii) generate additional oxygen vacancies that serve as redox-active sites, and (iii) facilitate faster ion transport by lowering diffusion barriers. Together, these effects minimize charge-transfer resistance and accelerate interfacial kinetics, thereby enabling rapid and reversible energy storage [35]. The nearly mirror-symmetric peak positions of the Nd/Gd-Co electrode further highlight its quasi-reversible nature, with minimal polarization losses even under dynamic cycling. The dominant electrochemical faradaic reaction can be represented as (4) [36]:

(4) $${Co}_{3}O_{4}+H_{2}O+{OH}^{-}⇌\;3CoOOH+e^{-}$$

where the multivalent cobalt centers cooperatively engage in electron–proton coupled redox processes. The structural robustness imparted by RE dopants ensures that these transitions proceed without compromising electrode integrity.

The electrochemical response of Co_3_O_4_-based electrodes is strongly dictated by their ability to sustain fast redox transitions at the electrode/electrolyte interface. In pristine Co_3_O_4_, charge storage originates from the Co^2+^/Co^3+^ couple, yet its kinetics are often restricted by sluggish ion transport and the tendency of nanoparticles to aggregate, thereby limiting the accessibility of redox sites. The introduction of RE elements fundamentally alters this scenario by simultaneously tuning the electronic structure and the surface architecture. The scan-rate dependent CV analyses provide a clear window into these kinetic processes. At lower sweep rates, 1–5 mV/s (Figure 4b), the electrodes have sufficient time to undergo near-equilibrium redox transitions, yielding sharp peaks and relatively narrow potential separations. As the scan rate increases to 10–100 mV/s (Figure 4c–f), all samples preserve their characteristic shapes, though with a predictable broadening of peak separation. This phenomenon indicates quasi-reversible behavior where polarization losses begin to accumulate due to a combination of charge-transfer resistance and slower electrolyte ion diffusion into the bulk of the material [37]. Importantly, the current response continues to scale with scan rate, reflecting the fact that the capacitive contributions remain largely surface-controlled even under rapid operation. A striking feature is the consistently superior performance of the Nd/Gd-Co electrode compared to its pristine and singly doped analogs. The CV traces of this electrode exhibit higher peak currents, signifying greater charge storage capability. The origin of this enhancement lies in the cooperative role of the two dopants: Nd^3+^ substitution promotes lattice strain and modifies the electronic environment of cobalt centers, while Gd^3+^ incorporation creates oxygen-vacancy-rich regions that act as additional redox-active sites [38,39]. When combined, these effects produce a conductive lattice with abundant electroactive centers and shortened ion-diffusion pathways [40]. Beyond electronic effects, the RE dopants exert a profound influence on the morphology of the oxide. The co-doped material develops a hierarchical structure composed of fine grains and interconnected flake-like features arranged in a porous network. This architecture is crucial for electrochemical performance: the porous channels allow rapid penetration of OH^−^ ions from the electrolyte, the enlarged surface area exposes more cobalt sites for redox reactions, and the interconnected framework ensures uninterrupted electron transport. On the other hand, in the singly doped electrodes, Nd-Co and Gd-Co, the dopant incorporation was less effective in balancing structural porosity and electronic conductivity, resulting in unfavorable surface coverage and reduced accessibility of active sites.

To further probe the redox kinetics of the prepared electrodes, the relationship between peak current density (*i*_p_) and the square root of the scan rate (*v*^1/2^) was examined. As shown in Figure 4g, all electrodes displayed a linear dependence, confirming that the charge storage behavior is predominantly governed by a diffusion-controlled mechanism, where electrolyte ion transport strongly influences overall electrochemical activity. The apparent diffusion coefficients (D) were subsequently quantified using the Randles–Sevcik relation (5) [41]:

(5) $$D=\frac{i_{p}}{2.69\times 10^{5}\times n^{3/2}\times A\times C\times v^{1/2}}$$

where i_p_ is the redox peak current density, *v*^1/2^ is the square root of scan rate, *n* is the number of electrons transferred, *A* is the electrochemically active surface area, *C* is the electrolyte concentration, and *D* represents the diffusion coefficient. The calculated values at 10 mV/s are summarized in Table 1 and graphically illustrated in Figure 4h for comparison. Among all compositions, the Nd/Gd-Co electrode yielded the largest diffusion coefficients for both anodic and cathodic processes, highlighting its superior ionic mobility and more favorable charge transport pathways. This enhancement can be directly linked to the synergistic effect of Nd^3+^ and Gd^3+^ dopants, which introduce lattice distortions and defect sites that facilitate electrolyte ion penetration while preserving continuous electron conduction networks. This integrated mechanistic insight aligns with prior studies demonstrating that dual doping strategies optimize electronic structure and ionic accessibility, outperforming single dopant systems [42]. In contrast, pristine Co_3_O_4_ and Gd-Co exhibited comparatively smaller diffusion coefficients. Their more compact and partially agglomerated morphologies restrict the accessibility of redox-active sites and slow down electrolyte diffusion, thereby limiting their kinetic response. Nd-Co showed moderate improvement over pristine Co_3_O_4_, yet still fell short of the Nd/Gd-doped system.

The charge storage mechanism of pristine and RE engineered Co_3_O_4_ electrodes was systematically evaluated using Dunn’s method, a well-established framework for distinguishing capacitive and diffusion-governed contributions. This approach is based on the power-law dependence of peak current (*i*) on scan rate (*ν*), expressed as (6) and (7) [43,44]:

(6) $$i\;={av}^{b}$$

(7) $$\log\left(i\right)=\log\left(a\right)+b\;log(v)$$

where *a* and *b* are adjustable parameters. The slope b of the *log*(*i*) against the *log*(*v*) plot (Figure 4i) provides mechanistic insight: values approaching 1 indicate capacitive-dominated storage governed by surface reactions, whereas values near 0.5 signify diffusion-limited charge storage involving ion intercalation and faradaic processes. In the present study, the b-values of all electrodes fell within the narrow range of 0.48–0.54 (Table 1), signifying that the charge storage process is predominantly diffusion-controlled, with the insertion of OH^−^ ions into the oxide lattice playing a central role.

**Figure 5:**
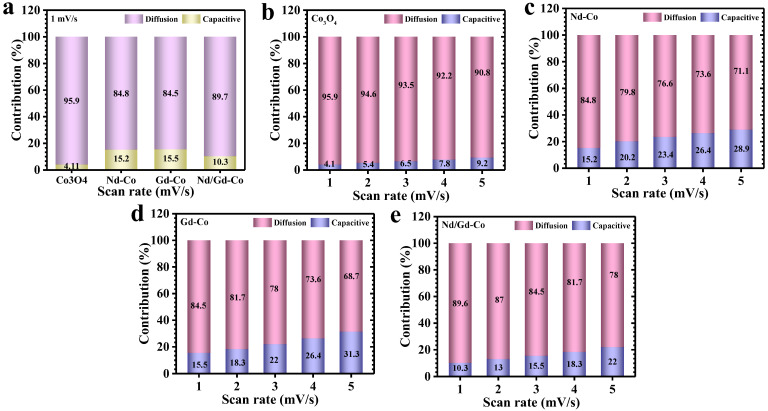
(**a**) Separation of capacitive and diffusion-controlled charge storage contributions at a scan rate of 1 mV/s for pristine Co_3_O_4_ and RE-Co_3_O_4_ electrodes, charge storage contributions at different scan rates (1–5 mV/s) for (**b**) Co_3_O_4_, (**c**) Nd–Co, (**d**) Gd–Co and (**e**) Nd/Gd–Co samples.

To further quantitatively resolve the capacitive and diffusion-controlled fractions, the current response was deconvoluted according to Equation (8) [45]:

(8) $$i\left(V\right)=k_{1}v{+k_{2}v}^{1/2}$$

where *k*_1_*ν* represents the capacitive component and *k*_2_*ν*^1/2^ reflects the diffusion-governed contribution. By plotting i(V)/$v^{1/2}$ vs. $v^{1/2}$, the relative values of k_1_ and k_2_ were extracted. At a scan rate of 1 mV/s (Figure 5a), the diffusion-controlled contributions were determined to be 95.9% for Co_3_O_4_, 84.8% for Nd-Co, 84.5% for Tb-Co, and 89.7% for Nd/Tb-Co electrodes. Among the systems, Nd-Co and Gd-Co exhibited the moderate reliance on diffusion-controlled processes. This behavior is attributed to the compact and aggregated surface morphology, where it hinders the ion transport into bulk domains and limits the diffusion-controlled participation. The Nd/Gd-doped electrode, in contrast, revealed a more balanced electrochemical character. Its hierarchical microstructure, composed of interconnected flake-like feature and fine grains, promotes dual functionality: extensive bulk ion transport combined with efficient surface redox activity. This synergy explains why the diffusion contribution is higher than in singly doped samples. By integrating abundant surface-accessible sites with continuous ion/electron transport pathways, the Nd/Gd configuration optimizes both kinetics and charge utilization. The effect of scan rate further reinforces these findings (Figure 5b–e). At higher sweep rates, all electrodes displayed an increasing capacitive contribution because bulk ion insertion becomes kinetically limited, forcing charge storage to shift toward surface-dominated processes. Conversely, at lower scan rates, diffusion-controlled redox activity overcomes, consistent with the measured b-values near 0.5 [46,47]. The ability of the Nd/Gd electrode to maintain balanced contributions across different rates highlights the role of RE co-doping in tuning both the intrinsic electronic structure and extrinsic morphology of Co_3_O_4_, thereby enabling superior performance under both energy-intensive (diffusion) and power-intensive (capacitive) operating regimes.

**Figure 6:**
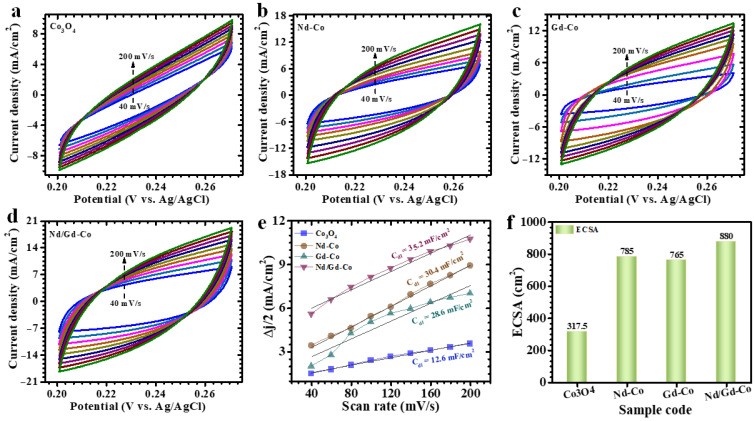
(**a**–**d**) CV measurements conducted at various scan rates within a non-faradaic region for all electrodes, (**e**) Plot of resulting current variations at different scan rates for assessments of double-layer capacitance (C_dl_) of the electrodes, and (**f**) The computed ECSA values for Co_3_O_4_, Nd–Co, Gd–Co and Nd/Gd–Co electrodes.

To assess the electrochemically active surface area (ECSA) of the Co_3_O_4_, Nd-Co, Gd-Co, and Nd/Gd-Co electrodes, CV was conducted at various scan rates confined to the non-faradaic region, as shown in Figure 6a–d. Utilizing this potential window ensured the measurement of pure capacitive current, eliminating interference from faradaic reactions. The resulting currents recorded at different scan rates were then used to evaluate the electrodes’ double-layer capacitance (C_dl_), presented in Figure 6e. The ECSA was subsequently determined using the following relation (9) [48]:

(9) $$ECSA=\frac{c_{dl}}{C_{s}}$$

where C_dl_ denotes the double-layer capacitance, and C_s_ is the specific capacitance value attributed to the material (0.04 mF cm^−2^; in 1M KOH). This approach offers a reliable means of quantifying the fraction of the electrode surface involved in charge storage, which is essential for understanding the energy storage behavior. Based on this method, the derived ECSA evaluations for Co_3_O_4_, Nd-Co, Gd-Co, and Nd/Gd-Co electrodes are approximately 317.5, 785, 765, and 880 cm^2^, respectively, as depicted in Figure 6f. The substantially higher ECSA of the Nd/Gd-Co sample indicates a highly porous and accessible surface, which supports effective ion transport and correlates directly with its improved electrochemical performance.

**Figure 7:**
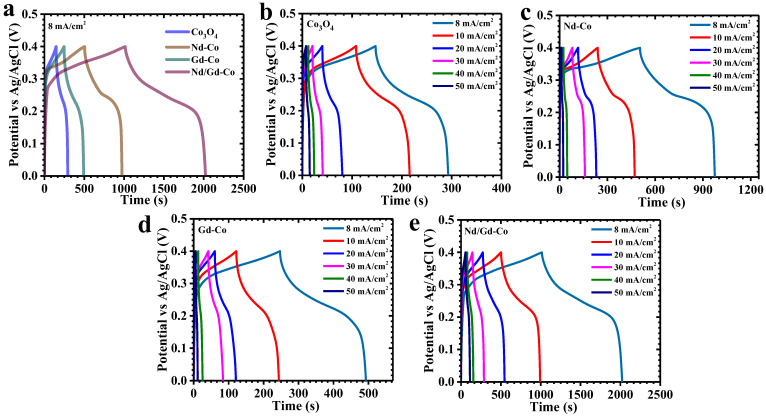
(**a**) GCD curves of pristine Co_3_O_4_ and RE-Co_3_O_4_ electrodes at 8 mA/cm^2^ current density, GCD profiles of (**b**) Co_3_O_4_, (**c**) Nd–Co, (**d**) Gd–Co, and (**e**) Nd/Gd–Co electrode at different current densities.

**Figure 8:**
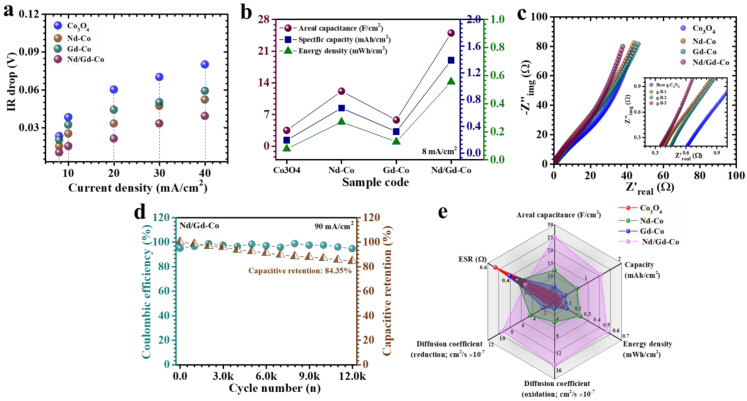
(**a**) IR-drop analysis illustrating internal resistance losses. (**b**) Plot comparing areal capacitance, specific capacity, energy density of pristine and RE-Co_3_O_4_ electrodes. (**c**) Nyquist plot of pristine and RE-Co_3_O_4_ electrodes. (**d**) Cyclic stability over 12,000 GCD cycles of Nd/Gd–Co sample. (**e**) Radar chart of the key electrochemical parameters for pristine Co_3_O_4_, singly doped Nd–Co and Gd–Co, and the co-doped Nd/Gd–Co electrodes.

To gain deeper insight into how RE incorporation influences the electrochemical response of Co_3_O_4_ electrodes, GCD and EIS analyses were carried out. Figure 7a presents the GCD curves of pristine and RE-doped Co_3_O_4_ electrodes recorded at 8 mA/cm^2^ within a 0–0.4 V window. All electrodes exhibited nonlinear charge–discharge profiles with distinct voltage plateaus, a characteristic of diffusion-limited faradaic activity associated with battery-type storage [49]. Among the investigated samples, the Nd/Gd-Co electrode displayed the most pronounced nonlinearity with a smoother and more stable discharge slope, signifying the dominance of pseudocapacitive behavior driven by reversible OH^−^ intercalation and surface redox reactions [49]. The considerably longer discharge time of the Nd/Gd-Co sample, compared to pristine Co_3_O_4_, Nd-Co, and Gd-Co, highlights its significantly improved charge-storage capacity enabled by the finely engineered nanostructure. Current-density-dependent GCD profiles (8–50 mA/cm^2^, Figure 7b–e) further confirmed these findings. Across the entire range, the discharge curves consistently retained redox-related voltage features already evident in CV, reinforcing the predominance of pseudocapacitive contributions. Nearly symmetric charge/discharge segments were observed for all electrodes, confirming high coulombic efficiency, minimal polarization losses, and stable ion diffusion kinetics. The Nd/Gd-Co electrode, in particular, exhibited an exceptionally small IR drop at the onset of discharge and maintained a highly symmetric profile across all current densities, reflecting superior electronic conductivity and highly reversible redox transformations. The charge-transport dynamics were further assessed by IR-drop analysis (Figure 8a). A systematic reduction in IR drop with decreasing current density was observed for all electrodes, signifying lower resistive losses at slower rates. Notably, the Nd/Gd-Co electrode consistently recorded the lowest IR-drop values across the entire current range, a clear indication of its minimized internal resistance and enhanced electrode–electrolyte interfacial kinetics.

To quantitatively evaluate electrochemical performance, areal capacitance (C_A_), energy density (ED), and power density (PD) were calculated using the following relations, specifically tailored for nonlinear pseudocapacitive GCD curves ((10)–(12)) [50,51]:

(10) $$C_{A}=\frac{I\times 2\times \int V\left(t\right)dt}{A\times {\left(∆V\right)}^{2}}$$

(11) $$ED=\frac{1}{2\times 3600}\;C_{A}\times {dV}^{2}$$

(12) $$PD=\frac{ED\times 3600}{T_{d}}$$

where *I* denotes the applied current, *∫V(t)dt* is the integrated area under the discharge curve, *A* is the geometric electrode area (cm^2^), Δ*V* is the potential window, and *T* is the discharge time. These expressions account for the intrinsic nonlinearity of faradaic GCD responses, thereby offering a realistic measure of the performance. At 8 mA/cm^2^, the areal capacitances of Co_3_O_4_, Nd-Co, Gd-Co, and Nd/Gd-Co electrodes were determined to be 3.5, 12.15, 5.8, and 25 F/cm^2^, respectively (Table 2, Figure 8b). The Nd/Gd-Co electrode thus achieved the highest capacitance, underscoring the effectiveness of dual RE integration. The outstanding performance of this electrode can be attributed to several synergistic factors: (i) the formation of a porous, high-surface-area nanostructure that provides a large density of electroactive sites; (ii) enhanced electronic conductivity arising from improved electron delocalization and stabilization of mixed Co oxidation states; and (iii) better electrolyte accessibility and shortened ion-diffusion paths facilitated by the interconnected flake–nanoparticle framework. Controlled doping levels also prevented Co_3_O_4_ particle agglomeration and suppressed particle restacking, thereby preserving the hierarchical architecture crucial for efficient ion transport and fast redox kinetics [52]. The dependence of capacitance and energy density on current density (Table 2) revealed a systematic decline with increasing current. This decrease is linked to restricted OH^−^ diffusion at high rates, which limits redox activity in the deeper layers of the electrode. Under such conditions, surface-accessible sites dominate, while bulk contributions diminish [53]. Despite this typical trend, the Nd/Gd-Co electrode retained 36% of its initial capacitance at 50 mA/cm^2^, demonstrating excellent rate capability and structural robustness under high-rate operation. Overall, the combined GCD analyses confirm that Nd/Gd co-doping delivers a well-optimized electrode architecture with minimized internal resistance, high electroactive surface exposure, and rapid ion/electron transport. This rationally engineered structure is the key to the superior charge-storage capability, rate performance, and stability of the Nd/Gd–Co electrode compared to pristine and singly doped counterparts.

EIS was performed at a fixed amplitude of 10 mV to investigate the charge-transport behavior of the Co_3_O_4_-based electrodes (Figure 8c). This analysis provides critical information on both resistive and capacitive elements governing electrode/electrolyte interactions. The Nyquist plots, which map the imaginary (−Z″) versus real (Z′) components of impedance, were used to extract the equivalent series resistance (ESR) as well as charge-transfer characteristics. The ESR corresponds to the intercept at the high-frequency region of the x-axis and reflects the intrinsic resistance of the electrode, including contributions from electronic conduction, electrolyte resistance, and contact interfaces [54]. As summarized in Table 1, the Nd/Gd-Co electrode exhibited the lowest ESR value of 0.26 Ω, markedly lower than those of pristine and singly doped counterparts. This pronounced reduction in resistance indicates superior electronic conductivity and more efficient ion/electron coupling across the electrode–electrolyte boundary. The improvement arises from the optimized and homogeneous microstructure generated through the controlled co-doping of Nd and Gd, which effectively reduces grain boundary barriers, enhances active site exposure, and stabilizes ion-diffusion channels. Such structural uniformity facilitates rapid ion migration and minimizes charge-transfer resistance, thereby accelerating faradaic kinetics. Literature correlates such microstructural homogeneity with improved electron delocalization and rapid ion transport kinetics. These factors collectively lower the resistive losses and facilitate faster redox reactions, contributing directly to the observed enhancements in capacitance and rate capability [55].

Long-term cycling stability at high current densities is a decisive factor in determining the practical feasibility of supercapacitor electrodes. To probe this aspect, the durability of the optimized Nd/Gd-Co electrode was evaluated at room temperature through continuous GCD cycling at 90 mA/cm^2^ current density for 12,000 successive cycles. The stability test was performed in a standard three-electrode configuration, where the Nd/Gd-Co electrode served as the working electrode, platinum was used as the counter electrode, and Ag/AgCl acted as the reference electrode. The electrolyte was 2 M KOH aqueous solution. The variation in capacitance retention and coulombic efficiency with cycle number is presented in Figure 8d. In the early cycles, the electrode exhibited a gradual increase in capacitance, which can be attributed to electrochemical activation and the progressive unblocking of internal pores that improved electrolyte penetration. Once activated, the electrode reached a steady state, maintaining highly stable performance with negligible declining during extended cycling. After 12,000 charge–discharge cycles, the Nd/Gd-Co electrode preserved ~84.35% of its initial capacitance, corresponding to a modest loss of ~16%, thereby highlighting its excellent long-term durability. Such retention underscores the structural robustness and chemical stability of the doped architecture, which can withstand repetitive ion insertion/extraction without significant mechanical degradation. The co-doped framework effectively accommodates volumetric fluctuations during cycling, suppresses pulverization, and maintains integrity of the redox-active sites. Equally important, the coulombic efficiency remained consistently high throughout the entire cycling test, reaching 94.46% even after 12,000 cycles. This exceptional reversibility reflects minimal parasitic reactions and highly efficient charge compensation during faradaic processes. The combination of stable capacitance retention and near-ideal coulombic efficiency validates the electrochemical resilience of the Nd/Gd-Co electrode, confirming its ability to deliver rapid and reversible redox reactions under demanding high-rate cycling conditions. Together, these results establish the Nd/Gd-Co system as a durable and reliable candidate for next-generation supercapacitor applications.

The radar chart displayed in Figure 8e provides a complete comparison of the key electrochemical parameters for pristine Co_3_O_4_, singly doped Nd–Co and Gd–Co, and the co-doped Nd/Gd–Co electrodes. The plotted axes incorporate critical performance indicators, including areal capacitance, energy density, power density, diffusion coefficient, and ESR. This multi-dimensional visualization clearly demonstrates the comprehensive advantages of the Nd/Gd–Co electrode. Unlike the pristine and singly doped counterparts, which show strong performance in only one or two metrics, the Nd/Gd–Co electrode achieves broad and balanced coverage across all parameters. Collectively, the radar plot underscores that the Nd/Gd–Co electrode does not simply excel in isolated aspects but achieves a synergistic integration of high capacity, rapid charge transport, low resistance, and durability within a single design. This balance validates the co-doping strategy as a rational and effective pathway for advancing next-generation supercapacitor electrodes.

In recent years, pristine and doped Co_3_O_4_ electrodes have been widely investigated for supercapacitor applications, with numerous strategies focused on improving capacitance, conductivity, and structural stability. Yet, while single RE doping has been reported to enhance certain electrochemical aspects, limited attention has been given to the synergistic effects of dual RE incorporation. The present study bridges this gap by systematically designing and evaluating Nd/Gd-Co electrodes, with their performance benchmarked against prior Co_3_O_4_-based systems. As summarized in Table 3 [56,57,58,59,60,61,62], the Nd/Gd-Co electrode was comparable to most reported Co_3_O_4_-based electrodes in terms of electrochemical parameters. This remarkable improvement originates from the co-doped hierarchical nanostructure, which integrates abundant electroactive sites, rapid ion diffusion channels, and reduced resistance within a single architecture. Incorporation of Nd and gadolinium Gd into Co_3_O_4_ offers distinct advantages for enhancing electrochemical performance. Nd doping induces lattice strain and modulates the electronic environment of cobalt sites, promoting enhanced charge carrier mobility and stabilizing multiple oxidation states vital for redox reactions. Gd doping primarily facilitates the formation of oxygen vacancies, increasing active site density and improving ionic diffusion pathways. The synergistic co-doping of Nd and Gd not only refines the crystal structure but also creates a highly porous morphology with improved electronic conductivity and electrolyte accessibility. This dual doping strategy leads to significant enhancements in capacitance, charge-transfer kinetics, and cycling stability, making Nd/Gd co-doped Co_3_O_4_ a promising electrode material for high-performance supercapacitors. Collectively, these results position Nd/Gd–Co as a superior electrode material and establish co-doping as an effective strategy for the rational design of next-generation high-performance supercapacitors.

The post-cycling XRD pattern (Figure S3a) retains all major diffraction peaks characteristic of the Co_3_O_4_ spinel phase (JCPDS No. 42-1467), with no detectable impurity phases or significant peak shifts, confirming the preservation of the primary crystal structure. Minor changes, such as increased background noise and slight reductions in peak intensity, suggest subtle lattice strain and limited surface restructuring due to repeated cycling. Further, surface morphology was examined using FESEM (Figure S3(b1–b3)), revealing a transition from relatively smooth, interconnected nanoparticles to a rougher, more fragmented texture after cycling. This morphological evolution, which includes the emergence of cracks and slightly increased irregularity is attributed to redox-driven surface oxidation, electrolyte-induced modification, and mechanical stress from continuous ion insertion/extraction. Such changes are expected in oxide-based electrodes and remain modest, indicating robust microstructural endurance despite prolonged use.

### 2.5. Electrochemical Performance of Asymmetric Supercapacitor Device

**Figure 9:**
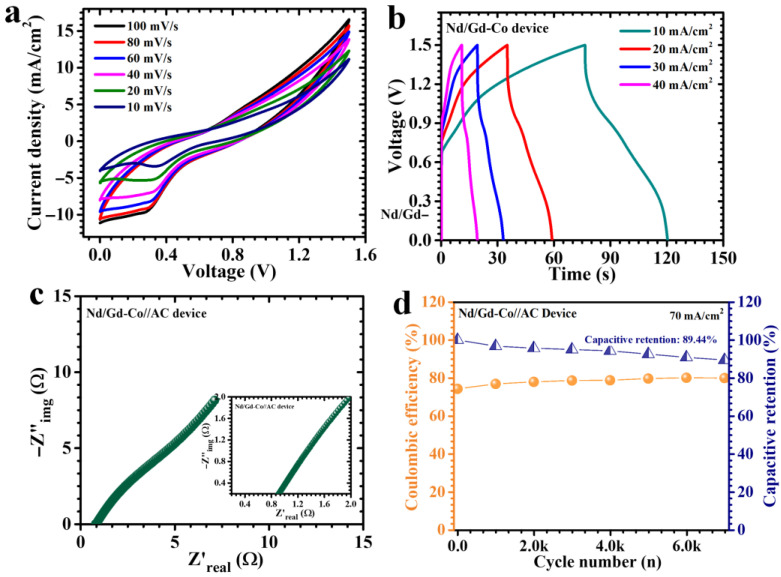
Electrochemical performance of the assembled asymmetric pouch-type supercapacitor device (APSD): (**a**) CV curves at various scan rates, (**b**) GCD charge–discharge profiles, (**c**) Nyquist plot indicating interfacial resistance characteristics, and (**d**) cycling stability with retention results after extended operation.

To demonstrate the practical utility of the RE-Co_3_O_4_ electrodes beyond the laboratory-scale three-electrode system, an APSD was fabricated and subjected to detailed electrochemical evaluation. In this configuration, the Nd/Gd-Co electrode served as the positive electrode, while AC, a material well known for its high surface area and excellent double-layer capacitance, was employed as the negative electrode. Both electrodes were directly supported on nickel foam current collectors, ensuring mechanical stability and good electrical contact. A sheet of filter paper soaked with 2 M KOH electrolyte acted as the separator, and the assembled pouch cell was carefully sealed to prevent external contamination and maintain stable operational conditions. The electrochemical performance of the APSD was assessed by CV, GCD, and EIS analyses. Figure 9a presents the CV profiles collected at different scan rates. The curves clearly confirmed a stable operational voltage window extending up to 1.5 V, which is considerably wider than that of typical aqueous supercapacitors and advantageous for boosting energy density. Across scan rates ranging from 10 to 100 mV/s, the CV curves maintained their characteristic shape with no noticeable distortion, while the current response increased proportionally with scan rate. This behavior reflects excellent electrochemical reversibility, efficient charge propagation, and robust electrode/electrolyte compatibility. The enhanced performance is attributed to the synergistic interplay between the Nd/Gd-Co positive electrode, which contributes pseudocapacitive faradaic reactions, and the AC negative electrode, which provides rapid double-layer charge storage. Further insights were obtained from GCD measurements at various current densities (Figure 9b). The discharge curves exhibited nonlinear profiles with distinct pseudocapacitive signatures, indicative of faradaic redox processes dominating the charge storage. At a current density of 10 mA/cm^2^, the APSD delivered an impressive areal capacitance of 140 mF/cm^2^, accompanied by an energy density of 0.044 mWh/cm^2^, and a power density of 1.8 mW/cm^2^ (Table 4). These values highlight the ability of the device to effectively combine high energy storage with rapid charge–discharge capability. The nonlinearity of the curves further corroborates the strong contribution of pseudocapacitive reactions from the Nd/Gd–Co electrode, which are well supported by the AC electrode.

The electrochemical impedance spectra (Figure 9c) provided complementary evidence of the device’s excellent charge-transport properties. The Nyquist plots revealed a small semicircle in the high-frequency region, corresponding to a very low equivalent series resistance (ESR) of 0.78 Ω. Such a low ESR value indicates minimal intrinsic resistance of the electrode materials, efficient ion migration across the separator, and excellent electrical conductivity within the electrode/electrolyte interface. Long-term durability, a critical parameter for real-world deployment, was also investigated through repeated charge–discharge cycling at a high current density of 70 mA/cm^2^. As shown in Figure 9d, the device maintained 89.44% of its initial capacitance after 7000 consecutive cycles, with only modest capacity decreasing despite the active testing conditions. Importantly, the coulombic efficiency remained consistently high, achieving 79.93% after extended cycling. The ability to sustain high efficiency and stable capacitance retention highlights the excellent reversibility of the faradaic reactions and the minimal side reactions occurring during prolonged operation. The outstanding cycling performance of the APSD can be directly linked to the structural and chemical stability of the Nd/Gd-Co electrode. The uniformly distributed, interconnected flake–nanoparticle morphology ensures maximum electrolyte accessibility, suppresses agglomeration of active particles, and prevents restacking-induced loss of surface area. The optimized RE dopant concentration plays a vital role in stabilizing this architecture. As a result, the Nd/Gd-Co electrode not only enhances initial electrochemical performance but also preserves long-term stability, making it a highly promising candidate for next-generation energy storage applications.

## 3. Conclusions

This study establishes RE co-doping as a powerful approach to enhancing the electrochemical performance of Co_3_O_4_ gels for supercapacitor applications. Morphological analyses revealed that co-doping produced a highly porous and interconnected framework, which promoted rapid electrolyte penetration and exposure of active sites. The Nd/Gd–Co electrode delivered an areal capacitance of 25 F/cm^2^ at 8 mA/cm^2^, significantly higher than that of pristine Co_3_O_4_ (3.5 F/cm^2^), Nd–Co (12.15 F/cm^2^), and Gd–Co (5.8 F/cm^2^). It also exhibited the lowest series resistance of 0.26 Ω, the highest ionic diffusion coefficients, and excellent stability with 84.35% capacitance retention and 94.46% coulombic efficiency after 12,000 cycles. When assembled into an asymmetric pouch-type supercapacitor, the Nd/Gd–Co//AC system achieved a 1.5 V operating window, an areal capacitance of 140 mF/cm^2^, an energy density of 0.044 mWh/cm^2^, and 89.44% retention after 7000 cycles. Taken together, these results clearly demonstrate that RE co-doping not only tailors the crystallographic and electronic structure of Co_3_O_4_ but also translates into practical device-level benefits. The synergistic effects of Nd and Gd incorporation yield electrodes with high capacitance, fast charge transport, and long-term durability, establishing Nd/Gd–Co gels as a promising class of materials for next-generation asymmetric supercapacitors.

## 4. Materials and Methods

### 4.1. Materials

All reagents were of analytical grade and used without further purification. Cobalt nitrate hexahydrate (Co (NO_3_)_2_·6H_2_O), neodymium nitrate hexahydrate (Nd (NO_3_)_3_·6H_2_O), gadolinium nitrate hexahydrate (Gd (NO_3_)_3_·6H_2_O), citric acid (C_6_H_8_O_7_), polyethylene glycol (PEG, average molecular weight ~6000), and ethanol were purchased from standard suppliers. Deionized water was used in all experiments.

### 4.2. Synthesis of Pristine and RE-Co_3_O_4_ Gels

**Figure 10:**
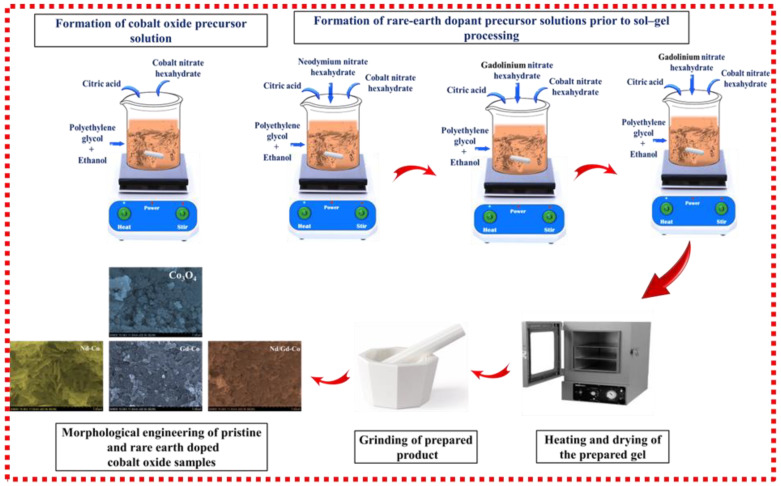
Schematic illustration of the sol–gel synthesis route used to prepare pristine Co_3_O_4_, Nd–Co, Gd–Co, and Nd/Gd–Co gels.

Co_3_O_4_ and RE-Co_3_O_4_ were synthesized by a sol–gel method. To initiate the process, a 0.2 M solution of Co (NO_3_)_2_·6H_2_O was prepared in ethanol under constant stirring until a clear and stable sol was formed. Subsequently, 0.1 M C_6_H_8_O_7_ was added to chelate the cobalt ions, thereby stabilizing the precursor solution. In the next step, PEG was introduced at a concentration of 0.23 g/mL, which acted as a structure-directing agent; as a result, the sol gradually developed a strong tendency toward gel formation. With continuous stirring, a homogeneous and transparent cobalt-based sol was obtained. In parallel, for the doped gels, 1 wt.% of Nd (NO_3_)_3_·6H_2_O was added for the neodymium-doped cobalt oxide (Nd-Co), 1 wt.% of Gd (NO_3_)_3_·6H_2_O was introduced for the gadolinium-doped cobalt oxide (Gd-Co), and a combination of 0.5 wt.% of Nd (NO_3_)_3_·6H_2_O with 0.5 wt.% of Gd (NO_3_)_3_·6H_2_O was used for the neodymium–gadolinium-doped cobalt oxide (Nd/Gd-Co). After the addition of these dopant precursors, the sols were stirred continuously until they dissolved completely, ensuring uniform distribution of the RE ions within the developing gel matrix. Such homogeneous mixing at the sol stage was crucial, as it effectively prevented phase segregation and allowed the subsequent gels to form with consistent composition throughout. Following precursor incorporation, the sols were gradually heated to 80 °C. At this stage, solvent evaporation occurred, and the sol underwent a smooth transition into a viscous polymeric gel. Thereafter, the obtained gels were dried at 120 °C for 12 h, which not only removed residual moisture but also consolidated the gel framework. Finally, the dried gels were calcined in air at 400 °C for 3 h with a heating rate of 5 °C/min. Figure 10 shows schematic of the sol–gel synthesis process for pristine and RE-Co_3_O_4_ gels.

### 4.3. Electrode Fabrication and Asymmetric Pouch-Type Supercapacitor Device (APSD) Assembly

The electrode fabrication process began with the mixing of Co_3_O_4_ and RE-Co_3_O_4_ powders, acetylene black, and polyvinylidene fluoride binder in a weight ratio of 80:10:10 (1 mg/cm^2^). The components were ground thoroughly to ensure a uniform distribution, and N-methyl-2-pyrrolidone (NMP, C_5_H_9_NO) was then added to form a viscous slurry. The resulting slurry (1 mg/cm^2^) was coated onto nickel foam substrates (1 × 1 cm^2^), selected for their high conductivity and porous structure that promote rapid ion transport and efficient electron pathways. The coated electrodes were dried at 80 °C to remove residual solvent and establish strong adhesion of the active material to the current collector. For the negative electrode, activated carbon was prepared under the same conditions to maintain comparable electrode architecture. An asymmetric pouch-type supercapacitor device was assembled by pairing the oxide-based electrode as the positive electrode with the activated carbon (AC) electrode as the negative electrode. The AC electrode was prepared by mixing activated carbon, carbon black, and PVDF in an 8:1:1 mass ratio (1 mg/cm^2^) to form a uniform slurry. This slurry was coated onto nickel foam and dried at 80 °C for 12 h to obtain the final electrode. A porous separator (soaked filter paper) was placed between the two electrodes, and aqueous 2 M KOH solution was introduced as the electrolyte. Electrochemical performance was initially examined for the individual electrodes in a three-electrode configuration to assess their intrinsic redox activity. Subsequently, the assembled APSD was tested under a two-electrode configuration to evaluate its practical performance in terms of capacitance, energy density, power density, and cycling stability.

### 4.4. Sample Characterization and Electrochemical Measurements

XRD analysis was conducted using a PAN-analytical instrument with Cu-Kα radiation to assess the structural characteristics and phase purity of the Co_3_O_4_ and RE-Co_3_O_4_ electrodes. The surface morphology and elemental composition of the electrodes were examined with field-emission scanning electron microscopy (FE-SEM, S4800 HITACHI, Ltd., Tokyo, Japan), equipped with an EDS. Prior to FE-SEM and EDS analysis, the samples were sputter-coated with a thin layer of platinum. XPS analysis was carried out with a K-Alpha instrument (Thermo Scientific, Eastleigh, UK) to investigate the chemical composition and valence states of the elements. Electrochemical tests were executed using a battery cycler (Biologic Instrument-WBCS3000, Bio-Logic, Gières, France) in a standard three-electrode setup, where Co_3_O_4_ and RE-Co_3_O_4_ served as the working electrode, platinum acted as the counter electrode, and Ag/AgCl was used as the reference electrode. A 2 M KOH solution was employed as the electrolyte for these measurements.

## Figures and Tables

**Table 1:** Estimated Diffusion coefficient, b-values, and series resistance values of pristine and RE-Co_3_O_4_ electrodes.

Sample Code	Diffusion Coefficient (cm^2^/s) × 10^−7^		b-Value	ESR (Ω)
	Oxidation	Reduction		
**Co_3_O_4_**	**1.2**	1.18	0.48	0.53
**Nd-Co**	4.85	4.34	0.53	0.33
**Gd-Co**	1.64	1.98	0.54	0.4
**Nd/Gd-Co**	15	9.32	0.52	0.26

**Table 2:** Evaluation of calculated areal capacitance, specific capacity, energy density, and power density values of pristine and RE-Co_3_O_4_ electrodes.

Sample Code	I (mA/cm^2^)	Areal Capacitance C_A_ (F/cm^2^)	Capacity (mAh/cm^2^)	Energy Density ED (mWh/cm^2^)	Power Density PD (mW/cm^2^)
**Co_3_O_4_**	8	3.500	0.194	0.078	1.92
	10	3.250	0.181	0.072	2.43
	20	2.250	0.125	0.050	4.50
	30	1.688	0.094	0.038	6.75
	40	1.250	0.069	0.028	8.33
**Nd-Co**	8	12.150	0.675	0.270	2.06
	10	7.375	0.410	0.164	1.78
	20	7.000	0.389	0.156	4.91
	30	7.000	0.396	0.158	7.22
	40	3.000	0.167	0.067	10.00
**Gd-Co**	8	5.800	0.322	0.129	1.89
	10	3.500	0.194	0.078	2.30
	20	3.375	0.188	0.075	6.43
	30	3.250	0.181	0.072	4.33
	40	1.250	0.069	0.028	7.69
**Nd-Gd-Co**	8	25.000	1.389	0.556	2.00
	10	16.750	0.931	0.372	4.91
	20	15.375	0.854	0.342	2.49
	30	12.750	0.708	0.283	7.08
	40	9.000	0.500	0.200	9.35

**Table 3:** Comparative literature summary table of Co_3_O_4_ electrodes.

Material	Current Density	Electrolyte	Specific Capacitance	Cycle Stability	Reference
CuO/Co_3_O_4_	2 A/g	3 M KOH	806.25 F/g	2000 cycles (99.75%)	[47]
Co_3_O_4_@NiMoO_4_	1 A/g	3 M KOH	600 C/g	8000 cycles (98.2%)	[48]
NiCo-phosphate	1 mA/cm^2^		3.69 F/cm^2^	1500 cycles (86.2%)	[49]
Co_3_O_4_	1 A/g	1 M KOH	464 F/g	10,000 cycles (87.2%)	[50]
r-GDYO/NiCo_2_S_4_	30 mA/cm^2^		3.09 F/cm^2^	5000 cycles (93.1%)	[51]
NiFe_2_O_4_-Co_3_O_4_@G			407 F/g	2000 cycles (91%)	[52]
Mg-Co_3_O_4_	10 mA/cm^2^	2 M KOH	136. mF	3000 cycles (93%)	[53]
**Nd/Gd-Co**	**8 mA/cm^2^**	**2 M KOH**	**25 F/cm^2^**	**12,000 cycles (84.35%)**	**This work**

**Table 4:** Calculated energy storage parameters of Nd/Gd-Co//AC asymmetric pouch-type supercapacitor device.

Sample Code	I (mA)	CA (mF/cm^2^)	C (mAh/cm^2^)	ED (mWh/cm^2^)	PD (mW/cm^2^)
**Nd/Gd-Co//AC device**	10	140	0.029	0.044	1.80
	20	137	0.029	0.043	3.25
	30	109	0.023	0.034	4.46
	40	84	0.017	0.026	5.66

## Data Availability

The data presented in this study are available on request from the corresponding author.

## Supplementary Materials

The following supporting information can be downloaded at: https://www.mdpi.com/article/10.3390/gels11110867/s1, **Figure S1:** (a–c) FE-SEM images (d–f) EDS and mapping analysis of Co_3_O_4_ electrode. **Figure S2:** (a–c) cyclic voltammetry of (a) Co_3_O_4_ N (b) Nd–Co (c) Gd-Co electrode at a scan rate of 1–5 mV/s. **Figure S3:** (a) XRD pattern of the Nd/Gd-Co electrode after 12,000 charge–discharge cycles, (b1–b3) FESEM images at different magnifications illustrating the morphological evolution of the electrode after long-term cycling. **Table S1.** Average lattice parameters and Scherrer crystallite sizes for pristine and RE-doped Co_3_O_4_ samples. **Table S2**. Fitted binding energies and area percentages for core-level peaks of Co 2p, Nd 3d, Gd 3d, and O 1s from high-resolution XPS spectra.
