# Supplementary material for: Engineering Rare Earth-Assisted Cobalt Oxide Gels Toward Superior Energy Storage in Asymmetric Supercapacitors
Source: Gels. 2025 Oct 29;11(11):867. doi: 10.3390/gels11110867 (PMC12651972; doi:10.3390/gels11110867)
Supplement: Supplementary file 1 [file gels-11-00867-s001.zip › gels-3921115-supplementary.pdf]

Supplementary Information

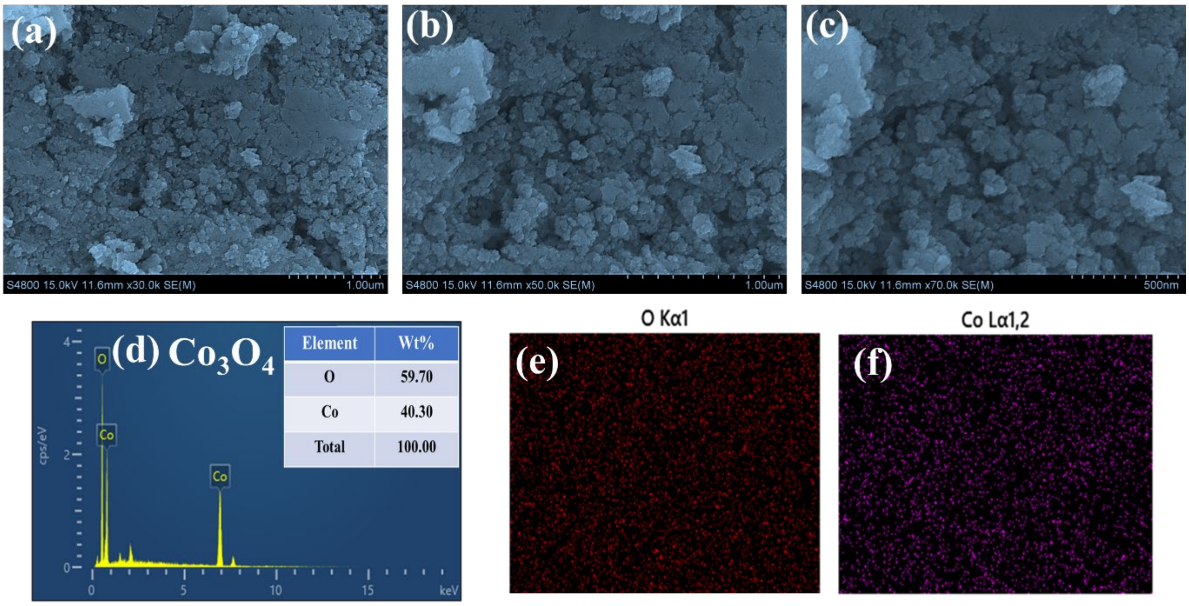

Figure S1: (a-c) FE-SEM images (d-f) EDS and mapping analysis of Co_3O_4 electrode.

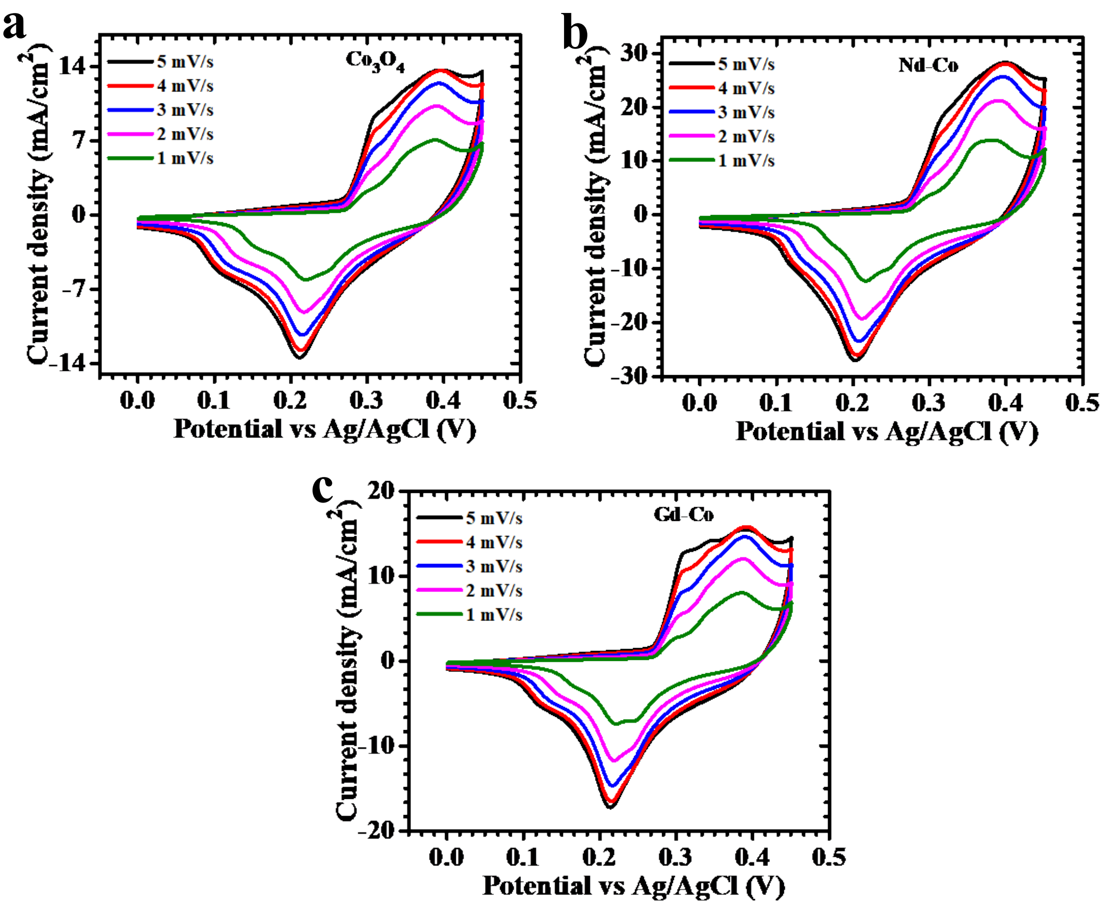

Figure S2: (a-c) cyclic voltammetry of (a) Co_3O_4 N (b) Nd–Co (c)Gd-Co electrode at a scan rate of 1-5 mV/s.

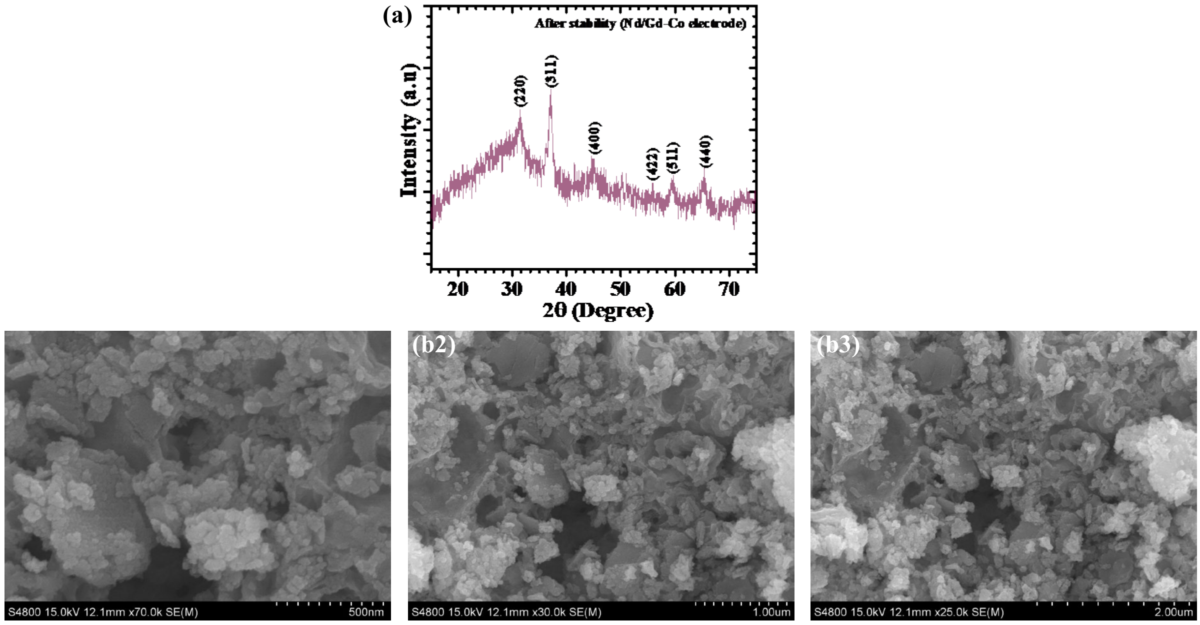

Figure S3: (a) XRD pattern of the Nd/Gd-Co electrode after 12,000 charge–discharge cycles, (b1–b3) FESEM images at different magnifications illustrating the morphological evolution of the electrode after long-term cycling.

**Sample code**	**Average lattice parameter a (Å)**	**Average crystallite size D (nm)**
**Co_3O_4**	≈ 8.087	≈ 79.46
**Nd-Co**	≈ 8.05	≈ 48.3
**Gd-Co**	≈ 8.047	≈ 39
**Nd/Gd-Co**	≈ 8.05	≈ 35.25

Table S1. Average lattice parameters and Scherrer crystallite sizes for pristine and RE-doped Co3O4 samples

**Element**	**Peak Assignment**	**Binding Energy (eV)**	**Area (%)**
**Co 2p**	Co $2p_{3/2}$	780.5	25.94568
**Co 2p**	Co $2p_{1/2}$	796.78	19.15848
**Nd 3d**	Nd $3d_{5/2}$	987.5	37.85171
**Nd 3d**	Nd $3d_{3/2}$	998.8	43.46322
**Gd 3d**	Gd $3d_{5/2}$	1186.8	31.33412
**Gd 3d**	Gd $3d_{3/2}$	1221.2	24.07498
**O 1s**	O^{2-}	529.8	54.14451

Table S2. Fitted binding energies and area percentages for core-level peaks of Co 2p, Nd 3d, Gd 3d, and O 1s from high-resolution XPS spectra.
